# Environmental levels of triclosan and male fertility

**DOI:** 10.1007/s11356-017-0866-5

**Published:** 2017-12-07

**Authors:** Joanna Jurewicz, Michał Radwan, Bartosz Wielgomas, Paweł Kałużny, Anna Klimowska, Paweł Radwan, Wojciech Hanke

**Affiliations:** 1 Department of Environmental Epidemiology, Nofer Institute of Occupational Medicine, 8 Teresy, 91-362 Lodz, Poland; 2 Department of Gynecology and Reproduction, “Gameta” Hospital, 34/36 Rudzka, 95-030 Rzgów, Poland; 3 Faculty of Health Sciences, The State University of Applied Sciences in Płock, 2 Dąbrowskiego Square, 09-402 Płock, Poland; 4 Department of Toxicology, Medical University of Gdańsk, 107 Hallera St, Gdańsk, Poland

**Keywords:** Environmental exposure to triclosan, Semen quality, DNA fragmentation, Sperm aneuploidy, Male fertility

## Abstract

Triclosan is a synthetic chemical with broad antimicrobial activity that has been used extensively in consumer products, including personal care products, textiles, and plastic kitchenware, although the exposure which is widespread evidence from human studies is scarce. Our study aims to investigate the relationship between triclosan exposure and male fertility. Triclosan (TCS) urinary concentrations were measured using gas chromatography coupled with tandem mass spectrometry in 315 men recruited from a male reproductive health clinic with normal sperm concentration (≥ 15 mln/ml) (WHO 2010) under 45 years of age. Participants were interviewed and provided a semen sample. TCS was detected in 84.13% of urine samples, with a median concentration of 2.83 μg/l (2.57 μg/g creatinine). A multiple linear regression analysis showed a positive association between the urinary concentrations of triclosan 50th–75th percentile and ≥ 50 percentile and percentage of sperm with abnormal morphology (*p* = 0.016 and *p* = 0.002, respectively). The study provides evidence that exposure to triclosan is associated with poorer semen quality. Future studies are needed to confirm these findings.

## Introduction

Triclosan (2,4,4 1-trichloro-2 1-hydroxy-diphenyl ether, TCS) is a broad-spectrum antibacterial and antifungal agent, widely used in personal care, household, veterinary, and industrial products (Rodricks et al. 2010).

The manufacture of TCS on a massive scale started in the 1970s, because it was considered to have low acute toxicity, and 20 years later, the compound reached the top 10 detected contaminants in American river (Halden 2014). In humans, ingestion and dermal absorption are the main routes of triclosan exposure (Sandborgh-Englund et al. 2006). After absorption in humans, TCS can be detected in urine, blood, milk, and plasma, the brain, adipose tissue, and liver (Wang and Tian 2015; Geens et al. 2012). Most of the absorbed doses are eliminated from the body within 24 h, mostly through with urine (Huang et al. 2016). Due to the rapid urinary excretion and the different exposure sources, the extracted concentration may vary over the day and between days (Lassen et al. 2013).

Recently, TCS has been suspected to be reproductive toxicant. Animal studies suggest that TCS exposure can decrease weights of the testes, sex organs, and sperm density (Kumar et al. 2008). It adversely affects the male reproductive system by disrupting steroidogenesis. Kumar et al. 2008 conducted in vitro studies in rodent Leydig cells and found that TCS depressed the synthesis of cAMP resulting in disruption of the steroidogenic cascade and leading to decreased testosterone synthesis (Kumar et al. 2009, Lan et al. 2015). The evidences of triclosan’s potential effects in human reproduction are still limited and conflicting. Zhou et al. 2016 measured urinary TCS concentration in men recruited through reproductive clinics and found decrease in sperm concentration, sperm count, the number of forward moving sperms, and the number of normally morphologic sperms (Zhou et al. 2016). Urinary levels of triclosan were negatively associated with reproductive hormones: inhibin B and LH (Den Hond et al. 2015). Additionally, urinary triclosan concentrations were inversely associated with early outcomes of in vitro fertilization-embryo transfer (IVF-ET) (top quality embryo formation and implantation rate) observed in women undergoing IVF-ET in China (Hua et al. 2017). Another study performed in China found no relationship between TCS exposure and idiopathic male infertility (Chen et al. 2013). Our aim was to study, for the first time, the association between environmental exposure to triclosan and male fertility, as indicated by semen quality (sperm concertation, motility, morphology, CASA parameters), sperm DNA damage, the level of reproductive hormones (FSH, testosterone, estradiol), and the total sperm disomy in adult men.

## Materials and methods

### Study participants and data collection

Males attending infertility clinic in Lodz, Poland, for diagnostic purposes with normal semen concentration of ≥ 15 mln/ml or (WHO 2010) between 2008 and 2011 were recruited. The details of the study population and recruitment were presented elsewhere (Jurewicz et al. 2014). Briefly, men under 45 years of age were eligible for study recruitment. All participants obtained and signed written informed consents prior to enrollment. Upon recruitment, a questionnaire was assigned to each participant to collect the data including demographic characteristics, occupational exposures, medical history, and lifestyle factors. Additionally, participants provided urine, saliva, blood, and semen samples on the same day of their clinic visit. Cotinine level in the saliva was measured to verify the smoking status as previously described (Jurewicz et al. 2014).

### Semen and reproductive hormones analysis

After the abstinence period, semen samples were generated via masturbation into polypropylene containers. Samples were liquefied at 37 °C for 20 min before analysis and semen parameters of sperm concentration, motility, and motion parameters were assessed according to World Health Organization (WHO 2010) guidelines. Analysis of the samples took place at the Andrology Laboratory with andrologists blinded to exposure status. The volume, pH, color, and viscosity were determined for each sample. Sperm morphology was quantified using strict Kruger criteria to classify men as having normal or below normal morphology (Jurewicz et al. 2014; Kruger et al. 1988). The detailed information about semen parameters analysis have been previously described (Jurewicz et al. 2014).

Sperm DNA damage was assessed based on sperm chromatin structure assay (SCSA) using flow cytometry (DAKO Galaxym DAKO, Denmark) (ASRM 2006, Evenson et al. 1999). The full details of the analysis of sperm DNA damage are presented elsewhere (Jurewicz et al. 2015).

To assess sperm aneuploidy, multicolor FISH analysis was performed using DNA probes specific for chromosomes 13, 18, 21, X, Y (AneuVysion DNA Probe Kit, VYSIS) as previously described (Radwan et al. 2015; Jurewicz et al. 2013). Based on this information about six types of chromosome disomies, total disomy was calculated.

Chemiluminescent Microparticle Immunoassay (ARCHITECT System; Abbott, Longford, Ireland) method was used to measure the levels of FSH, testosterone, and estradiol as previously described (Jurewicz et al. 2014).

### Urinary triclosan concentrations

Single, spot urine samples were collected and frozen at – 20 °C and sent to the laboratory in the Department of Toxicology, Medical University of Gdańsk. Analyses were performed using gas chromatography (Varian GC-450) coupled with tandem mass spectrometry (Varian 220-MS, ion-trap mass spectrometer).

Three milliliters of urine was placed in 10-ml screw-cap glass tube followed by 50 μl of mixed internal standard solution and 750 μl of freshly prepared acetate buffer (1 M, pH 5.0) containing 230 U of β-glucuronidase was added. Overnight incubation (at least 12 h) at 37 °C was performed. Then the sample was acidified with 450 μl of 80% formic acid and 3 ml of HEX:MTBE mixture (3:1, *v*:*v*) was added to the sample and tube was shaken for 10 min. After centrifugation, the organic layer was transferred into open glass tube and the extraction was repeated. Combined extracts were cleaned-up with 200 mg of MgSO4 and 10 mg of PSA by shaking in hands for 1 min. Then 5 ml of cleaned extract was transferred into new open glass tube and evaporated to dryness under stream of nitrogen at 35 °C. The residue was dissolved with 50 μl of BSTFA:TMCS (99:1) and derivatized for 30 min at 40 °C. One microliter of final extract was analyzed by GC-MS/MS. The limit of detection was 0.5 μg/L.

### Statistical analysis

Data management and statistical analysis were performed with R statistical software (ver.3) (R Core Team 2016). In case of the measurements below the limit of detection (LOD), 1/2 LOD was inputted. The proportion of samples below the limit of detection (LOD) was 15.87%. Descriptive statistics for subjects grouped by demographic characteristics were calculated, along with the distributions of urinary triclosan, and semen quality and reproductive hormone levels. Multiple least-squares linear regression models were used to quantify the associations of urinary triclosan (explanatory variables) with selected sperm quality measures and the concentrations of reproductive hormones as dependent variables. All exposure data were log transformed to obtain a normal distribution. Additionally, motility, percentage of sperm with abnormal morphology, DFI, and HDS were also subjected to shifted log transformation log(1 + x), to obtain more systematic quasi-normal distributions.

Negative binomial regression models were utilized for sperm disomy with the count of disomy outcome as the dependent variable and triclosan, with potential confounders as the independent variables. Disomy counts in binomial regression models were log transformed.

Creatinine-adjusted triclosan concentrations were categorized into four groups: first one consisted of values below limit of detection (LOD) to 25th percentile value, second—greater than the 25th percentile value to the median, third—greater than the median to 75th percentile value, while the fourth group consisted of values greater than the 75th percentile. Additionally, urinary concentrations of triclosan were presented as continuous variables and in categories below and above median.

Confounding factors in multiple regression models were predefined based on literature and biological consideration. The variables considered as the potential confounders included sexual abstinence (continuous), age (continuous), smoking (yes/no), past diseases (yes/no), and alcohol consumption (none or < 1 drink/week, 1–3 drinks/week, 4–7 drinks per week). Additionally, sperm disomy model was adjusted for sperm concentration and motility. A *p* value less than 0.05 was considered significant.

## Results

Demographic characteristics, semen quality parameters, level of reproductive hormones, and urine triclosan concentration are presented in Tables 1 and 2. A total of 315 men who attended infertility clinics for diagnostic purposes were enrolled in the study, mostly with higher (41.6%) or secondary (37.8%) education. The average age of the participants was 31.6 years. The average BMI was 26.8 ± 3.4 kg/m^2^. Most of the participants were nonsmokers (71%) (Table 1). Urinary concentrations of triclosan are presented in Table 2. The concentrations of total (free plus conjugated) triclosan were detected in 84.13% of samples at concentrations of 0.506–789.20 μg/l. The geometric mean and 95th percentile concentrations were 4.75 μg/l (4.27 μg/g creatinine) and 425.55 μg/l (362.21 μg/g creatinine), respectively. The mean semen concentration was 50.6 mln/ml (SD = 52.4, median 32.6) (Table 2). The mean percentage of DNA fragmentation index (DFI) was 16.5% (SD = 11.4%) (median 14%), high DNA stainability (HDS) was 7.7% (SD 3.9%) (median 7.16%), and total disomy was 1.72% (SD = 0.92) (median 0.82) (Table 2).

**Table 1:** Characteristics of the study population, *N* = 315

Characteristics	
Education, *n* (%)	
Primary and vocational	65 (20.6)
Secondary	119 (37.8)
Higher	131 (41.6)
Smoking determined by cotinine level, *n* (%)	
No	224 (71.1)
Yes	91 (28.9)
BMI (kg/m^2^), *n* (%)	
< 25	106 (33.7)
≥ 25	209 (66.4)
Mean (sd)	26.8 ± 3.4
Median (min–max)	27.6 (18.3–39.5)
Duration of couple’s infertility (years), *n* (%)	
1–2	120 (38.1)
2–3	104 (33.0)
3–5	45 (14.3)
> 5	46 (14.6)
Past diseases, which may have impact on semen quality, *n* (%)	
No	276 (87.6)
Yes	39 (12.4)
Abstinence [days], *n* (%)	
< 3	11 (3.5)
3–7	242 (76.8)
> 7	16 (5.1)
Missing data	46 (14.6)
Mean (sd)	5.0 ± 2.3
Median (min–max)	5.0 (0.0–20.0)
Age [years]	
Mean (sd)	32.14(4.2)
Median (min–max)	31.60 (22.01–44.3)
Alcohol use n(%)	
None or < 1 drink/week	104 (33.0)
1–3 drinks/week	163 (51.8)
Everyday	48 (15.2)

Past diseases which may have impact on semen quality—mumps, cryptorchidism, testes surgery, and testes trauma

*BMI* body mass index

**Table 2:** Semen quality, the level of reproductive hormones, and urinary concentrations of triclosan among study participants

Semen quality	Selected descriptive statistics									
	Min	25%	50%	75%	95%	Max	AM ± SD	GM ± SD	*N*	Frequency of detection
Main semen parameters										
Concentration (mln/ml)	15.0	22.5	32.6	85.8	125.0	360.0	50.6 ± 52.4	–	334	–
Motility (%)	4.0	46.0	55.0	66.0	83.0	99.0	55.7 ± 19.9	–	315	–
Sperm with abnormal morphology [%]	11.0	31.2	50.0	69.5	75.3	96.0	53.7 ± 23.9	–	315	–
CASA parameters										
VSL (μm/s)	14.9	37	43.1	50.7	61.4	77.1	43.6 ± 10.3	–	315	–
VCL (μm/s)	20.8	37.5	79.0	92.0	108.0	146.0	78.1 ± 16.8	–	306	–
LIN (%)	20.0	51.0	56.0	61.0	66.1	74.0	56 ± 6.4	–	315	–
DNA fragmentation index										
DFI	2.7	8.3	14.0	20.4	41.2	68.7	16.5 ± 11.4	–	262	–
HDS	0.7	5.2	7.2	9.3	13.5	30.7	7.7 ± 3.9	–	262	–
Total disomy	0	0.70	0.82	0.91	1.89	1.20	1.72 ± 0.92		220	
Triclosan concentration in urine										
Triclosan unadjusted (μg/l)	<LOD	0.84	2.83	21.49	425.55	789.20	57.71 ± 138.16	4.75 ± 9.92	315	84.13%
Triclosan CR-adjusted (μg/g creat)	<LOD	0.79	2.57	20.78	362.21	587.30	55.22 ± 121.78	4.27 ± 7.81	315	84.13%

*VSL* straight-line velocity, *VCL* curvilinear velocity, *LIN* linearity, *FSH* follicle-stimulating hormone, *HDS* high DNA stainability, *DFI* DNA fragmentation index, *AM* arithmetic mean, *GM* geometric mean, *SD* standard deviation

Triclosan concentration and the relevant male fertility outcomes are presented in Table 3. We observed that significant positive associations were observed between the urinary concentrations of triclosan 50th–75th percentile and percentage of sperm with abnormal morphology (*p* = 0.016) compared to urinary concentrations of triclosan < 25th percentile. Additionally, urinary concentrations of triclosan ≥ 50th percentile was also positively associated with percentage of sperm with abnormal morphology (*p* = 0.002) compared to urinary concentrations of triclosan < 50th percentile (Table 3).

**Table 3:** Triclosan concentration in urine and semen quality and the level of reproductive hormones-categories of urinary triclosan concentrations

		Triclosan				Triclosan		
		coef	95%CI	*p*		coef	95%CI	*p*
Sperm concentration	≤ 25th	ref			< 50th	ref		
	25th–50th	− 0.18	− 0.52; 0.15	0.29				
	50th–75th	0.03	− 0.31; 0.37	0.86				
	> 75th	− 0.05	− 0.39; 0.30	0.79	≥ 50th	0.08	− 0.16; 0.33	0.49
	cont	− 0.02	− 0.08; 0.05	0.57				
Motility	≤ 25th	ref			< 50th	ref		
	25th–50th	3.10	− 3.25; 9.46	0.34				
	50th–75th	1.92	− 4.52; 8.35	0.56				
	> 75th	1.39	− 5.09; 7.86	0.67	≥ 50th	0.10	− 4.48; 4.68	0.97
	cont	0.21	− 1.03; 1.45	0.73				
% of sperm with abnormal morphology	≤ 25th	ref			< 50th	ref		
	25th–50th	− 0.91	− 8.46; 6.65	0.81				
	50th–75th	9.37	1.74; 16.99	*0.016*				
	> 75th	6.84	− 0.84; 14.52	0.08	≥ 50th	8.57	3.14; 13.99	*0.002*
	cont	1.44	− 0.05; 2.92	0.06				
VCL	≤ 25th	ref			< 50th	ref		
	25th–50th	−0.93	− 3.71; 1.86	0.51				
	50th–75th	0.68	− 2.14; 3.49	0.64				
	> 75th	1.86	− 0.97; 4.68	0.20	≥ 50th	1.73	− 0.28; 3.73	0.09
	cont	0.19	− 0.35; 0.74	0.49				
VSL	≤ 25th	ref			< 50th	ref		
	25th–50th	−0.25	− 3.56; 3.07	0.89				
	50th–75th	1.85	− 1.50; 5.20	0.28				
	> 75th	1	− 2.37; 4.37	0.56	≥ 50th	1.55	− 0.83; 3.93	0.20
	cont	0.19	− 0.45; 0.84	0.55				
LIN	≤ 25th	ref			< 50th	ref		
	25th–50th	0.37	− 1.68; 2.42	0.72				
	50th–75th	1.14	− 0.93; 3.21	0.28				
	> 75th	−0.53	− 2.61; 1.55	0.61	≥ 50th	0.12	−1.35; 1.60	0.87
	cont	−0.01	− 0.41; 0.39	0.97				
Total disomy	≤ 25th	ref			< 50th	ref		
	25th–50th	1.05	0.88; 1.23	0.89				
	50th–75th	1.02	0.80; 1.29	0.85	≥ 50 th	1.15	0.22; 1.17	0.85
	> 75 th	1.18	0.33; 3.28	0.77				
	cont	1.22	0.44; 1.33	0.68				
DFI	≤ 25th	ref			< 50th	ref		
	25th–50th	0.12	− 0.16; 0.41	0.40				
	50th–75th	− 0.19	− 0.48; 0.1	0.20				
	> 75 th	− 0.06	− 0.34; 0.21	0.65	≥ 50th	− 0.18	− 0.39; 0.03	0.10
	cont	− 0.03	− 0.08; 0.02	0.24				
HDS	≤ 25th	ref			< 50th	ref		
	25th–50th	− 0.89	− 2.8; 1.02	0.36				
	50th–75th	− 0.57	− 2.49; 1.35	0.56				
	> 75th	− 0.71	− 2.55; 1.12	0.44	≥ 50th	− 0.25	− 1.64; 1.13	0.72
	cont	− 0.01	− 0.35; 0.33	0.94				

Statistically significant at the level 0.05. Multivariate model adjusted for sexual abstinence, age, smoking, alcohol consumption, past diseases, and creatinine. Total disomy model additionally adjusted for sperm concentration and motility. Italic values indicate significance level 0.05 was used for statistical intereference.

*coef* ß coefficient, *cont* continuous variable, *FSH* follicle-stimulating hormone, *HDS* high DNA stainability, *DFI* DNA fragmentation index, *TES* testosterone

## Discussion

In this study, we investigated the association between environmental exposure to triclosan and male fertility. We demonstrate, for the first time, the associations between triclosan exposure and different semen quality parameters (sperm concertation, motility, morphology, CASA parameters), sperm DNA damage, the level of reproductive hormones (FSH, testosterone, estradiol), and the total sperm disomy in adult men. The results of the present study suggest that urinary levels of triclosan increase the percentage of sperm with abnormal morphology. These findings suggest that environmental exposure to triclosan may affect semen quality.

The results are in agreement with the study performed by Zhu et al. 2016 where the environmental exposure to triclosan was inversely associated with the number of normally morphologic sperm, whereas no association between the level of triclosan with other semen parameters (sperm concentration, motility, CASA parameters), sperm DNA damage, sperm disomy and the level of reproductive hormones was observed. On the other hand, in a study performed in Belgium, TCS level was associated with decrease in the level of inhibin B and LH (Den Hond 2015). Those hormones were not analyzed in our study.

The biological mechanism of the impact of triclosan on semen quality is unclear but in vitro studies have demonstrated that TCS can bind with low affinity with estrogen and androgen receptors and to act as their agonist, antagonist or to result in no action (Witorsch 2014). It adversely affects the male reproductive system by disrupting steroidogenesis. Kumar et al. 2008 conducted in vitro studies in rodent Leydig cells and found that TCS depressed the synthesis of cAMP resulting in disruption of the steroidogenic cascade and leading to decreased testosterone synthesis. Forgacs et al. 2015 found that high doses of TCS inhibited testosterone synthesis but only rhCG-induced synthesis, while basal testosterone production remained unaffected. Kumar et al. 2009 carried out an in vivo study in rodent Leydig cells and found that higher doses of TCS caused a significant decrease in testis weight and sex accessory tissues. Another finding was the downregulation of testicular levels of mRNA for cytochrome P450scc, cytochrome P450c17, 3β-HSD, 17β-HSD, StAR, AR, and a decreased in vitro activity of testicular steroidogenic enzymes. They also reported decreased levels of serum LH, FSH, cholesterol, pregnenolone, and testosterone. All these findings were followed by decreased semen production (Kumar et al. 2009).

The urinary concentrations of triclosan in our study was lower compared to American level GM = 4.74 and 16.2 μg/l, respectively (NHANES 2009). Whereas compared to European countries, the level was similar to Belgium (GM = 2–3 μg/l) (Den Hond et al. 2015) and Spain (GM = 6.1 μg/l) (Casas et al. 2011) and higher compared to China (GM = 1.12 μg/l) (Zhu et al. 2016).

Triclosan and other nonpersistant chemicals have a short half-life; they are rapidly metabolized and could be totally excreted from the body in less than 24 h after exposure (Sandborgh-Englund et al. 2006). In our study, only one, spot urine sample was collected, so the misclassification of TC exposure is possible. However, stable, habitual lifestyle, and activity patterns may impact on stable exposure to triclosan and may represent individual’s general exposure level. Additionally, studies of temporal variability in urinary excretion of TCS have indicated reasonable temporal consistency in triclosan excretion (Lassen et al. 2013). Also, Pollack et al. 2016 demonstrated fair to good reliability for one urine sample for TCS. Second, since this is a cross-sectional study, the nature of this study does not allow the determination of causality of the observed associations.

The present study was conducted among men from infertility clinic, so it may limit the generalization the results to the general population. To limit this disadvantage, we recruit only men with normal semen parameters according to WHO classification (WHO 2010) (Jurewicz et al. 2014).

This is the first study, which assesses the semen quality, sperm DNA measures, level of reproductive hormones, and the total disomy in one study. Additionally, the total sperm disomy and sperm DNA damage were not analyzed in relation to triclosan exposure. A detailed questionnaire performed among study participants allowed for control of confounding factors. Also, the smoking status was confirmed by analyses the level of cotinine in saliva.

In conclusion, we have investigated the association between exposure to triclosan and male fertility measured by semen quality parameters, sperm DNA damage, total sperm disomy, and the level of reproductive hormones. We observed that triclosan exposure can increase the percentage of sperm with abnormal morphology. These findings provide one of the first evidence of adverse effects of triclosan exposure on male fertility. The results of the current study support the hypothesis that endocrine-disrupting chemicals are important factors for declining male semen quality. Those findings need to be confirmed in future studies.
